# Effects of atropine and propranolol on lung inflammation in experimental envenomation: comparison of two buthidae venoms

**DOI:** 10.1186/1678-9199-19-8

**Published:** 2013-04-09

**Authors:** Hadjer Saidi, Sonia Adi-Bessalem, Djelila Hammoudi-Triki, Fatima Laraba-Djebari

**Affiliations:** 1 USTHB, Faculty of Biological Sciences, University of Sciences and Technology Houari Boumedienne, BP 32 El Alia, Bab Ezzouar, 16111, Algiers, Algeria

**Keywords:** Scorpion venoms, Cytokines, Lung inflammation, Acetylcholine, Atropine, Propranolol

## Abstract

**Background:**

Previous works had shown that scorpion venom induced neurotransmitter elevation and an inflammatory response associated with various anatomo-pathological modifications. The most dangerous scorpions species in Algeria responsible for these effects are *Androctonus australis hector* (Aah) and *Androctonus amoreuxi* (Aam).

**Results:**

Comparison of the physiopathological effects induced by the two venoms showed differences in the kinetic of cytokine release and in lung injury.

The lung edema was only observed in response to Aah venom and it was correlated with cell infiltration. In order to better understand the involved mechanism in inflammatory response, we used two antagonists, atropine (non-selective muscarinic antagonist) and propranolol (β adrenergic antagonist), which lead to a decrease of cell infiltration but has no effect on edema forming.

**Conclusion:**

These results suggest another pathway in the development of lung injury following envenomation with Aam or Aah venom.

## Background

Scorpion envenomation is a serious problem common to many countries. In Algeria the most dangerous scorpion species are *Androctonus australis hector* (Aah), *Buthus occitanus tunetanus* (Bot) and *Androctonus amoreuxi* (Aam). Scorpion venoms are known to stimulate the autonomic nervous system simultaneously with release of tissue and medulla catecholamine [1-4]; induce an inflammatory response characterized by increase of cytokines, prostaglandines, leukotrienes, and platelet activated factor (PAF) in sera associated with inflammatory cell infiltration in tissues, especially lung [5-8].

Lung edema is the main cause of death after scorpion stings [9-11]. Its pathogenesis could be due to a non-cardiogenic effect following activation of inflammatory cascade and/or due to a cardiogenic effect [6,12-15]. Catecholamine may induce pulmonary edema via both hemodynamic and inflammatory mechanisms, by augmenting the IL-6 level [16]. The autonomic effects on inflammation are not restricted to catecholamine since the use of muscarinic antagonists may prevent some of the underlying cellular inflammatory responses in the lungs in addition to reducing smooth muscle contraction and mucus secretion [17,18]. Furthermore, the muscarinic antagonist, atropine significantly reduces neutrophil influx in lungs [19]. These polynuclear cells migrate into the lungs as a direct response to various proinflammatory stimuli and might contribute to many disorders such as acute respiratory distress syndrome (ARDS) [20,21].The present study is designed to investigate the mechanism by which venoms of two scorpions, found in Algeria and belonging to the same genus *Androctonus*, lead to lung inflammation by using atropine and propranolol.

## Methods

### Biological materials

#### Venoms

Lyophilized venoms of *Androctonus australis hector* and *Androctonus amoreuxi*, with respective LD_50_ of 0.85 and 0.75 mg/kg, were obtained from the Pasteur Institute of Algeria.

#### Animals

Male NMRI mice (20 ± 2 g), provided by the Pasteur Institute of Algeria, were used for all experiments. The animals were kept under controlled environment and received food and water *ad libitum*. The experimental protocol was in accord with the guidelines for the care of laboratory animals published by the European Union.

#### Non-biological materials

Chemical products and reagents used in these experiments were purchased from Sigma (USA), Merck (Germany) or Panreac (Spain). Pharmaceutical products were acquired from other firms, the atropine sulfate from Renaudin (France) and the propranolol from AstraZeneca (France).

#### Effect of *Androctonus amoreuxi* venom on cytokine levels

Groups of mice were injected by subcutaneous (s.c.) route with a sublethal dose of Aam venom dissolved in saline solution; control mice received 0.2 mL of saline solution alone. Mice were bled at several moments and sera were separated and stored at -20°C.

Cytokines were measured by specific sandwich ELISA, using cytokine Amersham kits for IL-1β, IL-6, and IL-10 according to the manufacturer’s instructions. Binding of biotinylated monoclonal antibodies was detected using streptavidin-biotinylated horseradish peroxidase complex and 3, 3’, 5, 5’ tetramethylbenzidine (TMB). Samples were quantified by comparison with standard curves of recombinant mouse cytokines. The lower limits of detection were 3 pg/mL (IL-1β), 7 pg/mL (IL-6) and 12 pg/mL (IL-10).

#### Effects of venoms on lung tissue

The effects of a sublethal dose of the two venoms, Aam and Aah, in the presence or absence of antagonists on lungs were evaluated by: estimation of myeloperoxydase activity as a marker of neutrophilia and histological study. Atropine sulfate (1 mg/kg) was injected intraperitoneally (i.p. route) 30 minutes before venoms and propranolol (0.1 mg/kg) was injected by the same route at two moments, 15 minutes before and 15 minutes after venom administration.

#### Myeloperoxydase activity

Three hours after envenomation by Aam or Aah venom, the removed lungs were homogenized in Tris–HCl buffer 50 mM, pH 6.6, then centrifuged at 6000 rpm for 30 minutes. The first supernatant (S1) was conserved at 4°C and the second supernatant (S2) was recovered after three freeze-thaw cycles of the pellet followed by its centrifugation at the above mentioned buffer conditions, rate and duration. One hundred microliters of S1 and 100 μL of S2 were added to 300 μL of chromogene substrate (0.167 mM O-dianisidine prepared in Tris–HCl 50 mM; pH 6.6 and H_2_O_2_ 8.8 mM) and the resulting mixture was read at the absorbance of 460 nm after one minute of incubation at room temperature.

#### Histological study

Lungs were fixed in 4% formaldehyde for 48 hours at room temperature, dehydrated in ethanol, cleared in xylen and embedded in paraffin. Histological sections (3-μm thick) were cut and stained with hematoxylin-eosin (H&E) for microscopic examination (Motic Digital Microscope PAL system).

#### Statistical analysis

The obtained data were expressed as mean ± SD and analyzed by, ANOVA with the significance level defined as p < 0.05.

## Results

### Effect of Aam and Aah venoms on cytokine release

**Figure 1:**
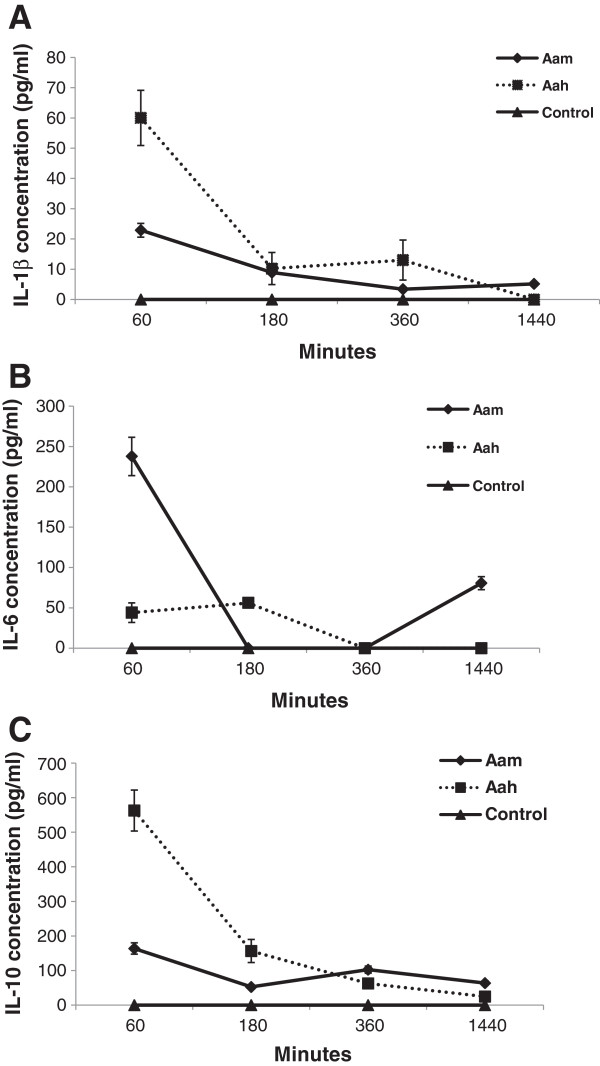
**Kinetic of cytokines release in sera following Aam or Aah injection.** (**A**) IL-1β, (**B**) IL-6, (**C**) IL-10 *p < 0.05, **p < 0.01, ***p < 0.001, NS: not significant, compared to control.

In the present study, the sera of mice envenomed by Aam displayed an increase of proinflammatory cytokines (IL-1β and IL-6). The comparison between Aam and Aah showed that the IL-1β level was more important in response to Aah venom (60 ± 12 pg/mL) versus (22.85 ± 2.15 pg/mL) (Figure 1 – A), while the maximum release of IL-6 was detected 60 minutes after Aam injection (237.66 ± 20.5 pg/mL), 180 minutes after administration of Aah venom (56 ± 2.89 pg/mL) followed by a significant elevation at 1440 minutes only in mice envenomed with Aam venom (Figure 1 – B).

In addition to the production of proinflammatory cytokines, the Aam and Aah venoms induced a significant release of an anti-inflammatory cytokine (IL-10). A biphasic profile was observed in response to Aam, the first peak measured at 60 minutes (162 ± 16.35 pg/mL), the second one at 360 minutes (104 ± 10.22 pg/mL), whereas only one peak was observed at 60 minutes (562.5 ± 59.3 pg/mL) in response to Aah venom (Figure 1 – C). Elevation of this cytokine was also reported in mice envenomed with *Centruroides noxius* venom [22].

### Myeloperoxydase activity

**Figure 2:**
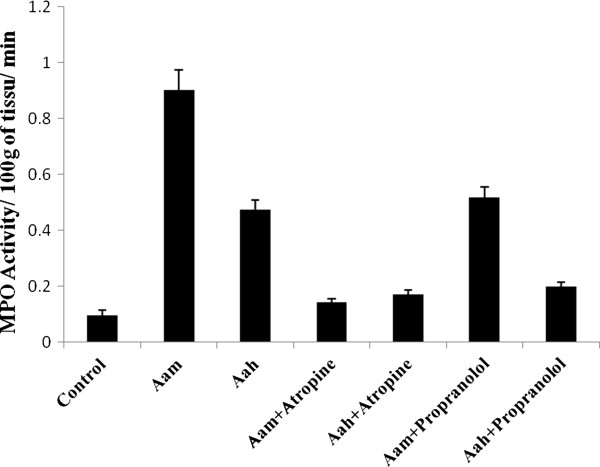
**Effect of *Androctonus amoreuxi *and *Androctonus australis hector *venoms on myeloperoxydase activity in the presence or absence of atropine (At) and propranolol (Pr).** *p < 0.05, **p < 0.01, **p < 0.001.

Rapid accumulation in lungs of neutrophils in response to any proinflammatory stimulus is one of the first recognizable events in the pathogenesis of many pulmonary diseases [23,24]. Neutrophil recruitment into lungs was evaluated by myeloperoxydase activity in this study, the two venoms induced neutrophil infiltration with more pronounced effect when Aam venom was injected (0.902 ± 0.071) versus (0.474 ± 0.033) (Figure 2).

The increase of pulmonary MPO activity was also observed in mice envenomed with Aah and *Tityus serrulatus* venoms [25,26].

Antagonists’ effectiveness in reducing neutrophil influx depended on the venom injected; in comparison to propranolol, atropine significantly prevented neutrophil recruitment in the presence of Aam venom and showed the same effect in response to Aah venom (Figure 2).

### Lung histology

**Figure 3:**
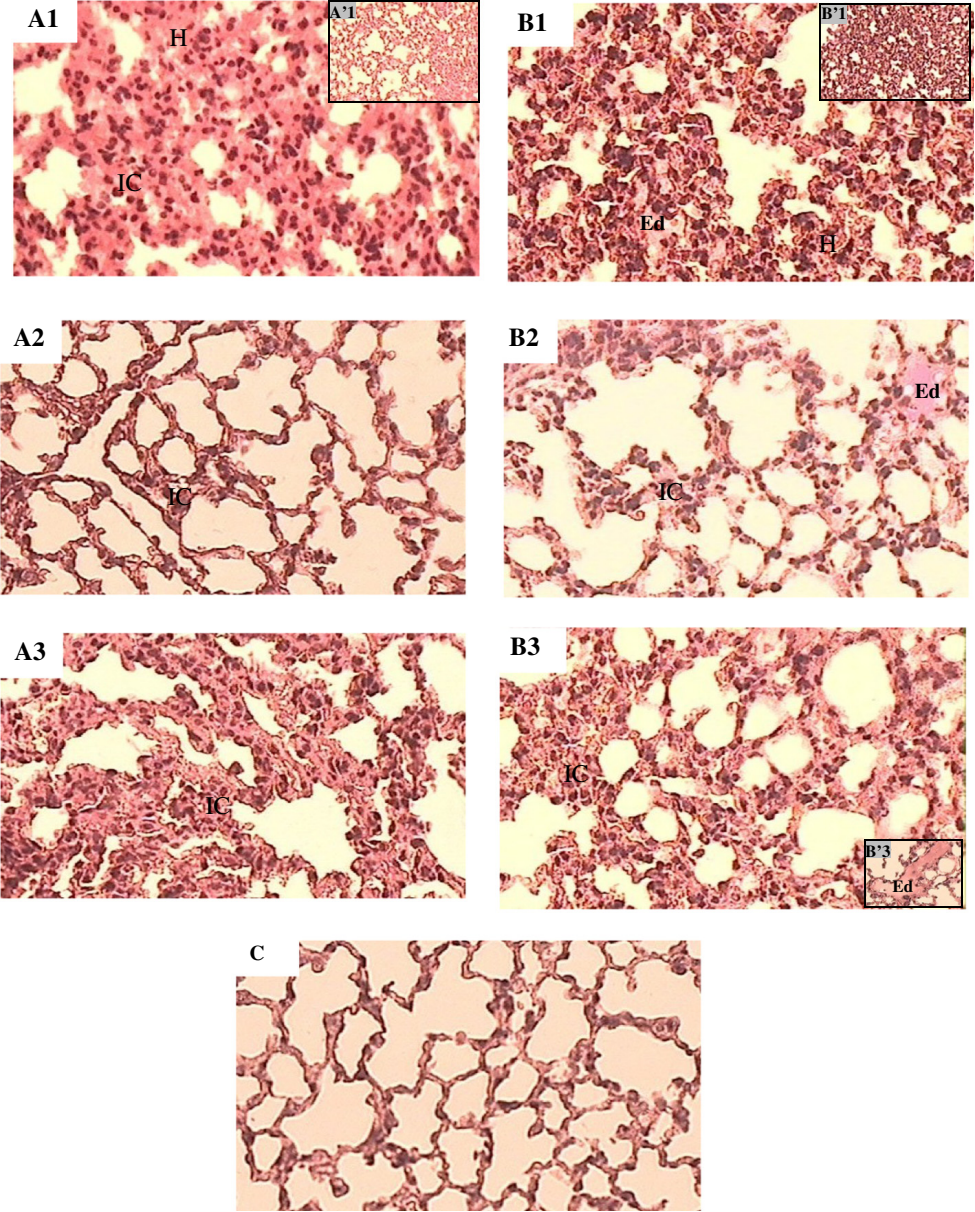
**Effects of *Androctonus amoreuxi *and *Androctonus australis hector *venoms on pulmonary parenchyma.** (**A1** and **B1**) Magnification of 400×, (**A’1** and **B’1**) magnification of 100×, in the presence of atropine (**A2, B2**) or propranolol (**A3**, **B3** and **B’3**), (**C**) control injected with saline. A: alveolus, E: edema, H: hemorrhage, IC: inflammatory cells, Hematoxylin-Eosin.

Lung tissue micrographs from mice envenomed by Aam or Aah showed hemorrhage, thickening of the inter-alveolar septa with a high accumulation of inflammatory cells (Figure 3 – A1, A’1, B1, B’1). These effects are more pronounced in response to Aah venom which showed, in addition to these anatomopathologic modifications, some edematous area (Figure 3 – B1).

Administration of atropine, a non-selective muscarinic antagonist or propranolol, a β adrenergic antagonist prior to the venom administration showed that atropine is more effective than propranolol at preventing inflammatory cell influx (Figure 3 – A2, B2, A3, B3). The lung edema observed in response to Aah venom was augmented in mice pretreated by the two antagonists (Figure 3 – B2, B’3).

## Discussion

Several studies emphasized the relevance of proinflammatory cytokines in the pathophysiological manifestations of scorpion envenomation and showed their correlation with severity [27-30].

The results of the present study showed that Aam and Aah venoms induced the release of proinflammatory cytokines IL-1β and IL-6. This finding is in agreement with previous studies that indicate an increase in circulating inflammatory cytokines after envenoming with several scorpion venoms such as *Tityus serrulatus*, *Tityus discripans, Centruroides noxius*, *Leiurus quinquestriatus quinquestriatus* and *Buthus occitanus*[7,22,27-29,31-33].

Cytokine levels differed significantly, with Aam venom inducing an earlier increase of IL-6 compared to Aah venom. This result can be explained by the high early level of anti-inflammatory cytokine (IL-10) in response to Aah venom. Indeed IL10 is known to play a modulatory role, down-regulating multiple aspects of immune and inflammatory responses through the regulation of the proinflammatory cytokines (IL-1β, IL-6 and TNF-α) [34,35].

The discrepancy in neutrophil influx observed in response to the Aam and Aah venoms may be attributable to their different levels of IL-6 release. This result is supported by previous data which showed that proinflammatory cytokines are responsible for leukocyte recruitment by inducing the elevation of chemokines and expression of adhesion molecules such as ICAM-1 and VLA-4 in endothelial cells [36]. The increase of chemokines might result in the binding of acetylcholine to muscarinic receptors [37,38].

Administration of atropine or propranolol prior to envenomation reduced neutrophilia in the lungs. This result suggests an inflammatory effect of both muscarinic and β adrenergic stimulation, as reflected in the ability of each antagonist to reduce neutrophil recruitment; which depends on the venom injected. Atropine was more effective than propranolol in preventing neutrophil influx following Aam injection. This observation is probably related to the amount of neurotransmitter released with a predominance of cholinergic stimulation in the presence of Aam venom.

This cholinergic predominance could explain in part the absence of an edematous area in lungs of mice envenomed by Aam venom, since the binding of Ach to muscarinic receptors decreases cardiac contraction, whereas excessive catecholamine release might give rise to left ventricular dysfunction that, in turn, may form lung edema [14,15,39-43]. Lung edema observed in pheochromocytoma patients or induced experimentally by catecholamine was prevented by pretreatment with α adrenergic blockers [16,44-47]. However our study showed that pulmonary edema subsequent to Aah venom was not reduced by propranolol or atropine. A similar effect was observed following propranolol administration in patients with pheochromcytoma [48]. Ismail [49] explained the effect of atropine by the accentuation of arterial hypertension.

The present comparison of lung micrographs between Aam and Aah also showed that an edematous area is observed only in response to Aah venom which induced a more important leukocyte infiltration in alveolar walls. These data are supported by previous studies which reported that edema formation in scorpion envenomation is attributable in part to activation of the inflammatory cascade and the release of lipid-derived mediators of inflammation, including PAF, leukotrienes and prostaglandins secreted after activation of mast cells by neuropeptides such as substance P [6,12,13].

The influx of inflammatory cells into pulmonary parenchyma was reduced by atropine and propranolol. These results are similar to other studies which ascribed inflammatory effects to β adrenergic stimulation, thus indicating that most inflammatory cells express functional muscarinic receptors and showed that atropine administration inhibits the migration of leucocytes towards the site of inflammation, and blocked increase of leucocytes in splenic venous blood in response to carbacholine [50-56].

## Conclusion

In conclusion, the comparative study of inflammatory response induced by Aam and Aah venoms showed not only the role of the neuroendocrine-immune axis in the development of lung inflammation with more important parasympathetic involvement in envenomed mice by Aam, but also that the role of atropine or propranolol in reducing inflammatory cells influx is independent of their effect on lung edema formation. The variability of venoms must be elucidated before an efficient treatment can be developed.

## Competing interests

The authors declare no conflicts of interest.

## Authors’ contributions

All authors collaborated in this work; they read and approved the final manuscript.

## References

[B1] Patterson RA Physiological action of scorpion venom AmJTrop Med Hyg 1960 9 4 410 414 10.4269/ajtmh.1960.9.410 14430926

[B2] Ismail M Osman OH Ibrahim SA el-Asmar MF Cardiovascular and respiratory responses to the venom from the scorpion Leiurus quinquestriatus East Afr Med J 1972 49 4 275 281 5053316

[B3] Ismail M Osman OH el-Asmar MF Pharmacological studies of the venom from the scorpion Buthus minax (L. Koch) Toxicon 1973 11 1 15 20 10.1016/0041-0101(73)90146-3 4725557

[B4] Ismail M Osman OH Gumaa KA Karrar MA Some pharmacological studies with scorpion (Pandinus exitialis ) venom Toxicon 1973 12 1 75 82 4818648 10.1016/0041-0101(74)90102-0

[B5] Freire-Maia L de Matos IM Heparin or a PAF antagonist (BN-52021) prevents the acute pulmonary oedema induced by Tityus serrulatus scorpion venom in the rat Toxicon 1993 31 9 1207 1210 10.1016/0041-0101(93)90137-8 8266352

[B6] De-Matos IM Talvani A Freire-Maia L Teixeira MM Evidence for the role of mast cells in the lung edema induced by Tityus serrulatus venom in rats Toxicon 2001 39 6 861 867 10.1016/s0041-0101(00)00225-7 11137547

[B7] Fukuhara YD Reis ML Dellalibera-Joviliano R Cunha FQ Donadi EA Increased plasma levels of IL-1beta, IL-6, IL-8, IL-10 and TNF-alpha in patients moderately or severely envenomed by Tityus serrulatus scorpion sting Toxicon 2003 41 1 49 55 10.1016/S0041-0101(02)00208-8 12467661

[B8] Adi-Bessalem S Hammoudi-Triki D Laraba-Djebari F Pathophysiological effects of Androctonus australis hector scorpion venom: tissue damages and inflammatory response Exp Toxicol Pathol 2008 60 4–5 373 380 18519162 10.1016/j.etp.2008.03.006

[B9] Campos JA Silva OA Lopes M Freire-Maia L Signs symptoms and treatment of severe scorpion sting in children Toxicon 1979 17 1 19

[B10] Goyffon M Vachon M Broglio N Epidemiological and clinical characteristics of the scorpion envenomation in Tunisia Toxicon 1982 20 1 337 344 10.1016/0041-0101(82)90240-9 7080047

[B11] Hering ES Jurca M Vichi FL Azevedo-Marques MM Cupo P ‘Reversible cardiomyopathy’ in patients with severe scorpion envenoming by Tityus serrulatus: evolution of enzymatic, electrocardiographic and echocardiographic alterations Ann Trop Paediatr 1993 13 2 173 182 7687114 10.1080/02724936.1993.11747642

[B12] de Matos IM Rocha OA Leite R Freire-Maia L Lung edema induced by Tityus serrulatus scorpion venom in the rat Comp Biochem Physiol 1997 118 2 143 148 10.1016/s0742-8413(97)00086-8 9440240

[B13] Matos IM Souza DG Seabra DG Freire-Maia L Teixeira MM Effects of tachykinin NK1 or PAF receptor blockade on the lung injury induced by scorpion venom in rats Eur J Pharmacol 1999 376 3 293 300 10.1016/S0014-2999(99)00382-9 10448890

[B14] Gueron M Margulis G Sofer S Echocardiographic and radionuclide angiographic observations following scorpion envenomation by Leiurus quinquestriatus Toxicon 1990 28 9 1005 1009 10.1016/0041-0101(90)90138-W 2260099

[B15] de Mazzei Dàvila CA Dàvila DF Donis JH de Bellabarba GA Villareal V Barboza JS Sympathetic nervous system activation, antivenin administration and cardiovascular manifestation of scorpion envenomation Toxicon 2002 40 9 1339 1346 10.1016/S0041-0101(02)00145-9 12220720

[B16] Rassler B Reibig C Briest W Tannapfel A Zimmer HG Catecholamine-induced pulmonary edema and pleural effusion in rats alpha- and beta- adrenergic effects Respir Physiol Neurobiol 2003 135 1 25 37 10.1016/S1569-9048(03)00062-4 12706063

[B17] Profita M Giorgi RD Sala A Bonanno A Riccobono L Mirabella F Muscarinic receptors, leukotriene B4 production and neutrophilic inflammation in COPD patients Allergy 2005 60 11 1361 1369 10.1111/j.1398-9995.2005.00892.x 16197467

[B18] Undem BJ Kollarik M The role of vagal afferent nerves in chronic obstructive pulmonary disease Proc Am Thorac Soc 2005 2 4 355 360 10.1513/pats.200504-033SR 16267362 PMC2713327

[B19] McQueen DS Donaldson K Bond SM McNeilly JD Newman S Bartona NJ Bilateral vagotomy or atropine pre treatment reduces experimental diesel-soot induced lung inflammation Toxicol Appl Pharmacol 2007 219 1 62 71 10.1016/j.taap.2006.11.034 17239416

[B20] Rolanld H Ingram JR Wilson JD, Braunwalf E, Isselbacher KJ, Petersdorf RG, Martin JB, Fauci AS, Root RK Syndrome de détresse respiratoire aigue de l’adulte Harrison- Principes de médicine interne 1992 Medicine Sciences Flammarion, Paris 1122 1125

[B21] Wagner JG Roth RA Neutrophil migration mechanisms, with an emphasis on the pulmonary vasculature Pharmacol Rev 2000 52 3 349 374 10977867

[B22] Petricevich VL Balance between pro- and anti-inflammatory cytokines in mice treated with Centruroides noxius scorpion venom Mediators Inflamm 2006 2006 6 1 11 10.1155/MI/2006/54273 PMC1775025 17392587

[B23] Worthen GS Nick JA Fishman AP Pulmonary diseases and disorders 1998 Mac Graw-Hill, New York 325

[B24] Nick JA Young SK Brown KK Avdi NJ Arndt PG Swatt BT Role of p38 mitogen activated protein kinase in murin model of pulmonary inflammation J Immunol 2000 164 4 2151 2159 10657669 10.4049/jimmunol.164.4.2151

[B25] Coelho FM Pessini AC Coelho AM Pinho VS Souza DG Arantes EC Platelet activating factor receptors drive CXC chemokine production, neutrophil influx and edema formation in the lungs of mice injected with Tityus serrulatus venom Toxicon 2007 50 3 420 427 10.1016/j.toxicon.2007.04.009 17532358

[B26] Adi-Bessalem S Mendil A Hammoudi-Triki D Laraba-Djebari F Lung immunoreactivity and airway inflammation: Their assessment after scorpion envenomation Inflammation 2012 35 2 501 508 10.1007/s10753-011-9338-0 21547500

[B27] Meki AR Mohey El-Dean ZM Serum interleukin-1β, interleukin-6, nitric oxide and α1 antitrypsin in scorpion envenomed children Toxicon 1998 26 12 1851 1859 9839669 10.1016/s0041-0101(98)00106-8

[B28] Hammoudi-Triki D Ferquel E Robbe-Vincent A Bon C Choumet V Epidemiological data, clinical admission gradation and biological quantification by ELISA of scorpion envenomations in Algeria: effect of immuno-therapy Trans R Soc Trop Med Hyg 2004 98 4 240 250 10.1016/S0035-9203(03)00062-2 15049463

[B29] Abdel Haleem AA Meki AR Noaman HA Mohamed ZT Serum levels of IL6 and its soluble receptor, TNF-α and chemokine RANTES in scorpion envenomed children: their relation to scorpion envenomation outcome Toxicon 2006 47 4 437 444 10.1016/j.toxicon.2005.12.008 16466762

[B30] Sofer S Gueron M White RM Lifshitz M Apte RN Interleukin-6 release following scorpion sting in children Toxicon 1996 34 3 389 392 10.1016/0041-0101(95)00136-0 8730932

[B31] D’Suze G Moncada S González C Sevcik C Aguilar V Alagón A Relationship between plasmatic levels of various cytokines, tumor necrosis factor, enzymes, glucose and venom concentration following Tityus scorpion sting Toxicon 2003 41 3 367 375 10.1016/S0041-0101(02)00331-8 12565760

[B32] Andrade MV Lisboa FA Portugal AL Arantes RM Cunha-Melo JR Scorpion venom increases mRNA expression of lung cytokines Comp Biochem Physiol A Mol Integr Physiol 2007 146 4 581 587 10.1016/j.cbpa.2006.01.031 16580239

[B33] Abdoon N Fatani A Correlation between blood pressure, cytokines and nitric oxide in conscious rabbits injected with Leiurus quinquestriatus quinquistratus scorpion venom Toxicon 2009 54 4 471 480 10.1016/j.toxicon.2009.05.009 19467253

[B34] Bogdan C Vodovotz Y Nathan C Macrophage deactivation by IL-10 J Exp Med 1991 174 6 1549 1555 10.1084/jem.174.6.1549 1744584 PMC2119047

[B35] Fiorentino DF Zlotnik A Mosmann TR Howard M O’Garra A IL-10 inhibits cytokine production by activated macrophages J Immunol 1992 147 11 3815 3822 1940369

[B36] Voronov E Apte RN Sofer S The systemic inflammatory response syndrome related to the release of cytokines following severe envenomation J Venom Anim Toxins 1999 5 1 19 23

[B37] Koyama S Rennard SI Robbins RA Acetylcholine stimulates bronchial epithelial cells to release neutrophil and monocyte chemotactic activity Am J Physiol 1992 262 4 Pt 1 L466 L471 1566862 10.1152/ajplung.1992.262.4.L466

[B38] Profita M Bonanno A Siena L Ferraro M Montalbano AM Pompeo F Acetylcholine mediates the release of IL-8 in human bronchial epithelial cells by a NFkB/ERK-dependent mechanism Eur J Pharmacol 2008 582 1–3 145 153 18242599 10.1016/j.ejphar.2007.12.029

[B39] Parthasarathy PR Venkaiah B Histopathological study on the effect of venom from the scorpion Heterometrus fulvipes Ind J Path Microbial 1986 29 2 155 158 3817946

[B40] Gueron M Ovsyscher I What is the treatment for the cardiovascular manifestations of scorpion envenomation? Toxicon 1987 25 121 124 10.1016/0041-0101(87)90170-X 3576632

[B41] Gueron M Ilia R Sofer S The cardiovascular system after scorpion envenomation a review Clin Toxicol 1992 30 2 245 258 10.3109/15563659209038636 1588674

[B42] Bucaretchi F Baracat EC Nogueira RJ Chaves A Zambrone FA Fonseca MR Comparative study of severe scorpion envenomation in children caused by Tityus bahiensis and Tityus serrulatus Rev Inst Med Trop Sao Paulo 1995 37 4 331 336 10.1590/S0036-46651995000400008 8599062

[B43] Gueron M Ilia R Non- cardiogenic pulmonary oedema after scorpion envenomation: a true antity? Toxicon 1996 34 4 393 395 10.1016/0041-0101(95)00147-6 8735237

[B44] Ferrari W Baggio G Guarini S Studies on epinephrine-induced lung edema in the rat. I. Selective a1-adrenoceptor involvement Arch Int Pharmacodyn Ther 1986 281 1 89 99 3019261

[B45] Tauzin-Fin P Hilbert G Krol-Houdek M Gosse P Maurette P Mydriasis and acute pulmonary oedema complicating laparoscopic removal of phaeochromocytoma Anaesth Intensive Care 1999 27 6 646 649 10631422 10.1177/0310057X9902700615

[B46] Van Iperen CE Giezen J Kramer WL Lips CJ Bartelink AK Acute dyspnoea resulting from pulmonary oedema as the first sign of a phaeochromocytoma Respiration 2001 68 3 323 326 10.1159/000050519 11416257

[B47] Rassler B Barth W Zimmer HG Transient pleural effusion in norepinephrine-stimulated rats Basic Res Cardiol 2001 96 5 265 273 10.1007/s003950170029 11605994

[B48] Sloand EM Thompson BT Propranolol-induced pulmonary edema and shock in a patient with pheochromocytoma Arch Intern Med 1984 144 1 173 174 10.1001/archinte.1984.00350130201036 6691755

[B49] Ismail M The scorpion envenoming syndrome Toxicon 1995 33 7 825 858 10.1016/0041-0101(95)00005-7 8588209

[B50] Sandberg G Leucocyte mobilization from the guinea pig spleen by muscarinic cholinergic stimulation Experientia 1994 50 1 40 43 10.1007/BF01992047 8293799

[B51] Chi DS Qui M Krishnaswamy G Li C Stone W Regulation of nitric oxide production from macrophages by lipopolysaccharide and catecholamines Nitric Oxide 2003 8 2 127 132 10.1016/S1089-8603(02)00148-9 12620376

[B52] Gosens R Zaagsma J Grootte Bromhaar M Nelemans A Meurs H Acetylcholine: a novel regulator of airway smooth muscle remodelling? Eur J Pharmacol 2004 500 1–3 193 201 15464033 10.1016/j.ejphar.2004.07.025

[B53] Gwilt CR Donnelly LE Rogers DF The non-neuronal cholinergic system in the airways: an unappreciated regulatory role in pulmonary inflammation? Pharmacol Ther 2007 115 2 208 222 10.1016/j.pharmthera.2007.05.007 17597218

[B54] Tan KS Nackley AG Satterfield K Maixner W Diatchenko L Flood PM Beta2 adrenergic receptor activation stimulates pro-inflammatory cytokine production in macrophage via PKA- and NF kappaB – independent mechanisms Cell Signal 2007 19 2 251 260 10.1016/j.cellsig.2006.06.007 16996249

[B55] Razani-Boroujerdi S Behl M Hahn FF Pena-Phillipides JC Sopori ML Role of muscarinic receptors in the regulation of immune and inflammatory responses J Neuroimmunol 2008 194 1–2 83 88 18190972 10.1016/j.jneuroim.2007.11.019 PMC2323336

[B56] Wessler I Kirkpatrick CJ Acetylcholine beyond neurons: the non-neuronal cholinergic system in humans Br J Pharmacol 2008 154 8 1558 1571 18500366 10.1038/bjp.2008.185 PMC2518461

